# Comparative Chemical Compositions of Fresh and Stored Vesuvian PDO “Pomodorino Del Piennolo” Tomato and the Ciliegino Variety

**DOI:** 10.3390/molecules23112871

**Published:** 2018-11-03

**Authors:** Nadia Manzo, Fabiana Pizzolongo, Giuseppe Meca, Alessandra Aiello, Nicola Marchetti, Raffaele Romano

**Affiliations:** 1 Department of Agricultural Sciences, Unit of Food Science and Technology, University of Naples Federico II, Via Università 100, 80055 Portici (NA), Italy; nadia.manzo@unina.it (N.M.) fabiana.pizzolongo@unina.it (F.P.), alessand.aiello@gmail.com (A.A.); rafroman@unina.it (R.R.); 2 Department of Chemistry and Pharmaceutical Sciences, University of Ferrara, 44121 Ferrara, Italy; mrcncl@unife.it; 3 Laboratory of Food Chemistry and Toxicology, Faculty of Pharmacy, University of Valencia, 46100 Burjassot, Spain

**Keywords:** Pomodorino del Piennolo del Vesuvio, cherry tomato, storage, chemical composition, antioxidants fractions, organic acids, volatile organic compounds

## Abstract

The Vesuvian Piennolo cherry tomato (*Lycopersicon esculentum Miller*) (PdP) is an old and typical variety grown in the Campania region (Italy). PdP is referred to as a long-storage tomato due to its thick and coriaceous skin that allows long post-harvest storage and it has been granted Protected Designation of Origin (PDO) status since 2009. In this study, the chemical composition, focusing in particular on organic acids, antioxidant molecules and volatile compounds, were investigated in PdP and compared to another typical variety in Campania, the Ciliegino tomato (CIL). Chemical characterization was evaluated for both the CIL and PdP varieties during storage in the same environmental conditions until deterioration of 50% of the fruits; deterioration occurred in PdP after 6 months and in CIL tomatoes after 1 month. The results demonstrated variation in the chemical profiles of both varieties with storage length. Particularly, the PdP variety appears richer in antioxidants compounds (i.e., chlorogenic acids and lycopene) and organic acids (i.e., glutamic and malic acids) than does CIL. Additionally, both varieties display different profiles of volatile bioactive compounds and they are differently influenced by the storage time. The results indicate a typical chemical composition of this long-storage tomato closely linked to the geographic origin area.

## 1. Introduction

**Figure 1:**
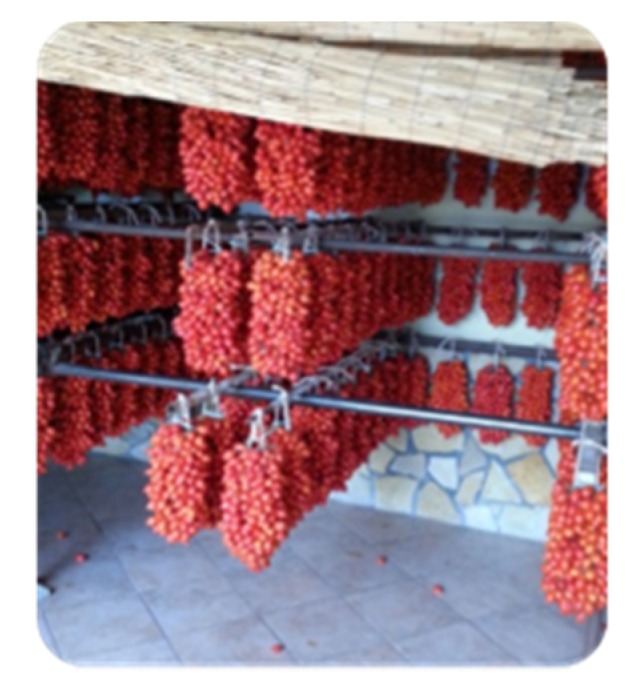
“Pomodorino del Piennolo”.

In Italy, tomato cultivation is characterized by many different traditional varieties that in most cases cover very limited areas. One of the most relevant is the Vesuvian Piennolo cherry tomato (PdP) from the Campania region. Its name is due to the ancient practice of ripening these tomatoes around a circled twine to form a large bunch, called a “piennolo”, that is stored hanging in dry, ventilated rooms (Figure 1).

The variety is registered in the “Register of the protected designations of origin and protected geographical indications” Reg. CE n. 1238/2009 [1] as published on the G.U. of January the 04^th^, 2010 [2]. The Protected Designations of Origin (PDO) Pomodorino del Piennolo del Vesuvio refers to tomatoes of ecotypes belonging to the *Lycopersicon esculentum Miller* species, locally known as “Fiaschella”, “Lampadina”, “Patanara”, “Principe Borghese” and “Re Umberto” and traditionally grown in specific areas near Mount Vesuvius.

According to the PDO regulation, protected fruits have to meet specific requirements such as colour, consistency, taste and an optical residual that must be a minimum of 6.5 °Brix. The total soluble solids content measured in Brix degrees is an important control parameter for tomato flavour, which is predominantly due to sugars. The main free sugars of commercial varieties of tomatoes are reducing sugars in particular, fructose has a large impact on sweetness perception [3].

Tomatoes are widely consumed in the Mediterranean region, and many varieties are cultivated for either fresh consumption or industrial processing. The whole fruit of tomatoes is an important source of bioactive compounds, including seeds and skin that contain approximatively 27% extractable lipids (predominantly oleic and linoleic acid) and approximatively 11% protein, respectively [4] The bioactive compounds have been associated with reduced risks of some types of cancer and other chronic diseases [5,6,7]. These beneficial effects have been mainly linked to lycopene and other carotenoids that represent the main antioxidant compounds found in fresh tomatoes and their processed products.

The main carotenoids present in tomatoes are lycopene and β-carotene. Lycopene is 80–90% of the total carotenoid content and it is responsible for the fruit’s red colour [8]. It has been shown to have a strong antioxidant activity and to exhibit the highest physical quenching rate constant for singlet oxygen. On the other hand, β-carotene is 7–10% of the total carotenoid content and it is of special interest due to its provitamin A activity [9]. Tomatoes represent, by far, the main source of dietary lycopene, whereas many other dietary sources contribute to the daily intake of β-carotene. However, the tomato fruit is a reservoir of other potentially healthy molecules, such as ascorbic acid, vitamin E and phenolic compounds, particularly flavonoids [10,11].

Moreover, other bioactive compounds can contribute to the antioxidant effects such as polyphenols. Several studies demonstrated that the positive effect of antioxidant-rich foods on health is seldom exerted by a single compound, or even by a single class of compounds but by the entire pool of antioxidant components interacting synergistically [12,13,14]. The antioxidant substance content of tomatoes depends on several factors such as the cultivar and fruit maturity, but also includes agronomic and environmental conditions during cultivation. Among these factors, the genotype is the most relevant for determining the pool of antioxidant compounds [15,16,17,18].

Delgadillo-Díaz et al. [19] studied the physico-chemical characteristics, content of antioxidant compounds and antioxidant capacity of the genotypes of creole tomatoes cv. Costoluto Genovese (*S. lycopersicum* L.) and wild (*S. pimpinellifolium* L.) grown in an aquaponic system (inorganically fertilized) and in soil (organically fertilized). The authors did not find differences in the total phenols and total flavonoids content between tomatoes harvested from aquaponic and soil systems. The total soluble solids, total carotenoids, and antioxidant capacity properties were higher in aquaponic tomatoes. No differences were due to the cropping system and genotype for antioxidant capacity.

Renna et al. [20] studied some biochemical parameters of the Regina tomato, and in particular three ecotypes (Monopoli, Fasano and Ostuni) were investigated for physical and chemical composition both at harvest and after three months of storage. The experimental results indicated that this tomato landrace has a qualitative profile characterized by high concentrations of tocopherols, lycopene and ascorbic acid, even after a long storage time, and a low average value of total soluble solids. The initial and post-storage contents of the bioactive compounds changed at different rates in each ecotype. Monopoli, having the highest content of tocopherol in fruits at harvest, had a reduction of approximatively 25% after 96 days of storage, while in the other two ecotypes, storage did not affect the tocopherol content. These results indicated that the unique and unmistakable features of this long-storage tomato depend on both natural (available technical inputs) and human (specific cultural practices) factors.

Raffo et al. [11] found that in the “Ciliegino” cherry variety tomato the average content of some classes of antioxidants is generally higher than that in normal-sized berries. Cherry tomatoes harvested at full ripeness showed the highest level of antioxidant activity and carotenoids in the water-insoluble fraction. On the other hand, no significant differences in the ascorbic acid content were observed at different ripening stages, whereas the main phenolics content and the antioxidant activity of the water-soluble fraction showed slight but significant decreases at later stages of ripeness.

To our knowledge only a few studies are available on PdP [21] investigating the carotenoid and flavonoid content in tomatoes from this cultivar. The studies found that PdP is a rich source of antioxidants, with lycopene and quercetin as the major components.

The aim of this study was to evaluate the main physical and chemical traits of PdP at harvest and after six mounts of storage and to compare the quality and nutritional traits of this ecotype to Ciliegino (CIL), another typical variety of cherry tomato in Campania.

## 2. Results and Discussion

### 2.1. Physical Chemical Parameters and Colour Measurement

The deterioration of 50% of fruits occurred after 6 months in PdP and after 1 month in CIL tomatoes. The chemical–physical parameters measured before and after storage are reported in Table 1. In particular, the pH values were significantly (*p* < 0.05) different between PdP and CIL, ranging from 4.13 to 4.45 for CIL1 and PdP6, respectively. For Piennolo tomatoes the pH values are not significantly different during storage, while for CIL the pH decreases (*p* < 0.05) after 1 month of storage and this is the lowest pH value that has been detected.

Regarding the total soluble solids (TSS), the levels of this parameter significantly increased after storage for both varieties. The lowest and highest TSS values were found in CIL0 and CIL1 with (i.e., 6.3 and 8.5 °Brix, respectively). The TSS parameter for PdP was higher than 6.5 °Brix, as requested by PDO regulation. Particularly, it raised from 6.9 to 7.9 °Brix during the storage.

The analysis outcomes concerning the dry matter evidenced quite close parameters for PdP0 and CIL0 (i.e., 7.2 and 7.7, respectively) at the beginning of the storage period, while for PdP6 and CIL1, the percentage values of dry matter were 8.4 and 9.55 respectively. The levels of dry matter significantly increased during storage by approximatively 1.2% and 2.0% in PdP6 and CIL1, respectively, and despite what was expected, the loss of moisture was remarkably larger in CIL after 1 month than in PdP after 6 months of storage.

The relative contents of reducing sugars such as fructose and glucose have a very important impact on the sweetness perception, and the two varieties analysed, PdP and CIL, revealed considerably different levels (i.e., 2.89 and 3.77%, respectively). Additionally, during storage the levels of this parameter increased in CIL.

The sodium chloride (NaCl) content in tomatoes is another parameter related to organoleptic characteristics, and the two varieties did not show significantly different values for fresh tomatoes. During storage the two types of cherry tomatoes behaved differently and in particular NaCl slightly increases in PdP6 (0.18% vs. 0.20%), while in CIL1, the relative amount of NaCl becomes approximatively 2.5 times larger (0.16% vs. 0.41%).

The last parameter analysed was the titratable acidity. The significance of this parameter is of concerns for microbiological fermentation, and its value has a strong influence on the quality of the final product. Citric acid is the most abundant acid in tomatoes and it is the main contributor to the total titratable acidity [22]. Particularly, the data reported in Table 1 show similar values for fresh tomatoes (0.53 and 0.43 for PdP0 and CIL0, respectively). Conversely, storage can affect this parameter differently for the two varieties. The acidity of PdP does not change significantly after 6 months of storage (0.53% vs 0.50%), while for CIL this parameter increases from 0.43% to 0.57% after 1 month.

### 2.2. Colour Measurement and Lycopene Content

Colour changes during tomato ripening have been studied using specific parameters, such as L*, a*, b* and Hue, through the Hunter colour scale values. The lightness factor L* and the a* values were constant during storage for both PdP and CIL (see Table 2). These parameters are correlated with the lycopene concentration [23] and they could also be related to the stability of lycopene itself during storage. The values of b* and a/b ratio were constant in PdP, while they significantly (*p* < 0.05) decreased in CIL during ripening, reflecting the loss of β-carotene in the latter case.

The hue, which is the actual colour (for example, red, yellow, blue, etc.), remained constant in PdP (0.64 and 0.67 in PdP0 and PdP6, respectively) and decreased from 1.00 to 0.67 in CIL.

Tomatoes are usually selected by consumers on the basis of appearance; hence colour is one of the most important quality factors that affect tomato appearance [24]. Tomato fruits can exhibit different external colours such as red, yellow, orange and pink as the result of both flesh and skin colours. Thus, a pink tomato may be due to colourless skin and red flesh, while an orange tomato may be due to yellow skin and red flesh [25]. Red tomato fruits as well as other types of Italian long-storage tomatoes are characterized by the presence of a thick and coriaceous skin. Therefore, it is possible that the external colour of red tomato fruits is strongly affected by their skin colour.

Among the carotenoids contained in tomatoes, lycopene is the most abundant, being present in a range from 6 to 540 mg/kg and depending on several factors related to cultural practices, variety and maturity [26]. Frusciante et al. [13] commented that the regular consumption of tomatoes has been associated with decreased risk of chronic degenerative diseases. Epidemiological findings confirm that the observed health effects are due to the presence of different antioxidant molecules such as carotenoids, particularly, lycopene, ascorbic acid, vitamin E and phenol compounds, mainly flavonoids.

In Table 2 are shown the lycopene amounts detected in the samples analysed. In particular, in PdP samples at 0 and 6 months of storage, the detected concentration of this bioactive compound was 73.52 and 43.32 mg/kg, respectively, whereas in CIL0 and CIL1 samples the concentrations of lycopene were 21.14 and 32.39 mg/kg, respectively. A more detailed examination of the data in Table 2 shows that the lycopene content detected in the CIL variety is statistically lower than that one detected in PdP. Additionally, during the storage period, the concentration of lycopene remained constant in CIL samples, while it significantly decreased from 73.52 to 43.32 mg/kg (44.05%) in PdP. Our findings in this study are in accordance with literature data [21], in which authors investigated the antioxidants composition of the PdP variety, and the lycopene concentration was found to be approximatively 78.6 mg/kg, which is very close to our result. Moreover, cell-based studies have suggested that synergistic effects among phytochemicals in tomatoes are responsible for their antioxidant activity, rather than single-molecule bioactivity. The authors demonstrated that the combination of low concentrations of carotenoids and polyphenols (i.e., not effective concentrations) resulted in a significant reduction of reactive oxygen species formation.

The data obtained by the same authors on lycopene are extremely comparable to those obtained in our study. Conversely, the data related to quercetin are ten times larger than our results.

In regards to relationship between colour and lycopene content, it is interesting to highlight that in some cases, deep red varieties of tomatoes (hybrids with so-called “high pigment”) can contain more than 180 mg of lycopene per kg of fresh weight [27], while some yellow varieties have been found to have only 5 mg/kg fresh weight [28]. Several authors reported the correlation between the skin colour indexes of tomatoes and the lycopene content, and they suggested equations to calculate and predict the lycopene concentration based on the skin colour [29]. According to these authors, the redder the skin colour, the higher the lycopene content in tomato fruits. These results suggest that the external colour of tomatoe fruits is strongly affected by their main morphological traits, especially possessing a thick and coriaceous skin, and suggest that for long-storage tomatoes, it may be difficult to find a correlation between skin colour indexes and lycopene contents.

### 2.3. Phenolic Composition

In Table 3 the phenolic compounds detected in the two varieties PdP and CIL, are reported at the two storage times considered above. The phenolic compounds contents detected in PdP0 and CIL0 were 38.54 and 29.63 mg/100 g, respectively. In the case of PdP6, the content of these bioactive compounds statistically decreased to 34.27 mg/100 g (11%), whereas for CIL1 the total phenols content increased statistically in comparison with the control experiment to 37.48 mg/100 g (20.9%).

Upon analysing the contents of the individual phenolic compounds detected, chlorogenic acid and rutin (quercetin 3-*O*-rutinoside) were the most abundant phenolic compounds evidenced in these vegetable matrices. In particular the rutin contents detected in PdP0 and CIL0 were 7.37 and 4.41 mg/100 g respectively, and significantly increased during ripening in both cultivars. The amounts were higher than those found by other authors [30] who reported an overall amount of 0.63 mg/100 g in green through red cherry tomatoes during vine ripening. This compound has been found to be the major flavonoid compound in ripened tomatoes in most studied cultivars due to its antioxidant properties and bioavailability to humans [31].

The chlorogenic acid content detected in the sample PdP0 was 10.30 mg/100 g, which was significantly higher (23.5%) than that found in CIL in both studied storage periods and higher than that in PdP6. Quercitin was detected in small amounts decreasing from 0.95 to 0.34 mg/100 g (35.7%) in PdP. The values detected are in accordance with other studies [21] that reported a value of 8.52 mg/kg FW (i.e., 0.85 mg/100 g FW) of quercitin in PdP. In the samples stored for 6 months and 1 month, the detected amount of this antioxidant decreased in both varieties and ranged from 0.34 to 1.87 mg/100 g, respectively.

### 2.4. Organic Acids

Table 4 lists the quantities of organic acids detected in the two varieties. In particular glutamic acid was the most abundant compound present in PdP, and the content of this compound ranged from 826.45 to 830.47 mg/100 g between the beginning of the storage time and after 6 months, respectively. Glutamic acid is a well-known compound that is able to influence tomato flavour. Additionally, many authors have highlighted the role of this compound in fruit acceptability and umami taste [32,33].

Considering the other organic acids in both tomato varieties, it is possible to underline that in PdP, the citric acid content increased from 404.14 to 441.66 mg/100 g (9%) during 6 months of storage. The variation of citric acid with storage time showed different trends for the CIL variety after 1 month, the amount of citric acid increased from 557.60 mg/100 g to 811.51 mg/100 g (31.3%). A reason for this can be the fermentative processes due to the natural microflora that characterize this food matrix.

PdP samples were also characterized by a high content of malic acid, which is considered an index of freshness and its concentration levels ranged from 580.48 to 492.62 mg/100 g at the beginning of storage and after 6 months, respectively. The results evidenced in our study are also in accordance with those reported by other authors [32].

The minor organic acids detected were succinic, oxalic and ascorbic acid. In particular, succinic acid is an important molecule involved in the citric acid cycle [34] and its amount ranged between 16.21 and 54.38 mg/100 g.

The quantity of oxalic acid in PdP was significantly lower than in CIL (see Table 4). This bioactive compound is important from a nutritional point of view because it forms insoluble salts with calcium and other divalent cations that produce a decrease in the bioavailability of these minerals [35].

Finally ascorbic acid showed a significant decrease during ripening in both PdP and CIL. The mechanisms of ascorbic acid losses could be due to multiple factors, such as oxidation, pH, moisture and temperature. However, ascorbic acid is relatively stable in tomatoes because of the acidic conditions found in this vegetable tissue [36].

### 2.5. Volatile Organic Compounds

Natural volatile organic compounds (VOC) play an important role in the aromatic component of tomato flavor. VOCs identified and quantified in PdP and CIL were grouped into 4 different classes, including aldehydes, alcohols, ketones and organic acids (Table 5).

Aldehydes were the most abundant VOC found in the two tomato varieties under investigation, and hexanal was the most abundant compound in both PdP and CIL at all times during the storage period (i.e., 55.79 and 100.22 mg/kg for PdP and 57.22 and 25.90 mg/kg for CIL, respectively). Additionally, important concentrations of heptanal and 2-hexanal have been detected. These are known as “green compounds”, as they impart a fresh, green character to the tomato aroma [37]. According to other authors [38] the main precursors of hexanal in tomato are leucine and β carotene and the main precursors of 2-hexenal are 2-isobutylthiazole and 6-methyl-5-hepten-2-one.

Considering the alcohols fraction in the control PdP sample, 1-hexanol was determined at 44.80 mg/kg and decreased to 9.13 mg/kg (80%) after 6 months storage. The concentration of this alcohol in CIL at both storage period ranged from 7.27 to 6.43 mg/kg, respectively. 1-Hexanol is a volatile compound derived from lipids via oxidation when cells are disrupted (like hexanal), and it imparts a mint flavour in tomatoes [39].

The main ketone present at the highest concentration was 6-methyl-5-hepten-2-one (i.e., 1.25 and 3.38 mg/kg in CIL0 and CIL1, respectively). This compound is a carotenoid-related compound and has a tomato-like flavour, musty aroma and fruity taste [39].

The last noticeable aspect of VOC in PdP and CIL is that PdP is characterized by a large concentration of 2-isobutylthiazole (i.e., 29.44 mg/kg). This compound is one of the most well-known thiazols and has a strong green odour that resemble tomato leaves, and it is considered fundamental in the development of proper tomato flavour [40].

A deep look at the results in Table 5 reveals that there is not clear correlation between the variation of VOCs and the storage time for both varieties. In some cases, specific volatile compounds, such as aldehydes, increased with storage in PdP, while in the cases of CIL, the same compounds decreased with storage.

## 3. Materials and Methods

### 3.1. Plant Material

Plant material consisted of two tomato varieties: Pomodorino del Piennolo (PdP) collected in Ercolano area (Naples, Italy) and Ciliegino (CIL) tomatoes purchased in Italian market. The two varieties were stored in the same environmental conditions until the deterioration of 50% of fruits that in PdP occurred after 6 months and in CIL tomatoes after 1 month. PdP after 0 and 6 months of storage (coded PdP0 and PdP6 respectively) and CIL after 0 and 1 month of storage (coded CIL0 and CIL1 respectively) were washed, chopped and submitted to successive analysis.

### 3.2. Determination of pH, Dry Matter and Total Soluble Solids

The pH was measured by using a pH-meter (Crison Basic 20, Crison Instruments S.A., Alella, Barcelona, Spain).

The determination of the dry matter (DM) was carried out by drying 3 g of homogenised sample in a stove at 70 °C, until the complete elimination of water. The content was calculated as percentage of total solids weight/ sample fresh weight.

The content of total soluble solids (TSS) was measured by using a refractometer Sper Scientific (Scottsdale, AZ, USA). The results were reported as Brix degree, which is defined as the concentration (%) of total soluble solids in solution when measured at 20 °C.

### 3.3. Determination of the Reducing Sugars

The reducing sugars were determined by Fehling’s method [41] on diluted and filtered samples. The parameter was calculated as percentage of reducing sugars weight/ sample fresh weight.

### 3.4. Determination of the Titratable Acidity

The total acidity was determined by titrating diluted sample with 0.1 M NaOH in the presence of phenolphthalein, until obtaining a persistent pink coloration [41]. The parameter was expressed as percentage of citric acid monohydrate weight/ sample fresh weight.

### 3.5. Colour Measurement

Hunter color scale values (L, a, b) were analyzed to determine the color change during storage.

Colour values were expressed as L* (whiteness or darkness), a* (redness/greenness) and b* (blueness/yellowness). Hunter scale parameters were determined using a Chroma Meter color analyzer (Minolta Chroma Meter Camera Co. Ltd., Osaka, Japan). The ratio a/b was also used to estimate total colour.

### 3.6. Determination of Lycopene

In agreement with Cucu et al. [42], 2 g of each sample were dissolved in 25 mL of hexane/acetone/ethanol 2:1:1 with 0.1% of butylated hydroxytoluene (BHT). The solution was mixed for 5 min with an Ultra-turrax (15000 rot/min). Then 15 mL of a saturated NaCl solution were added. The mix was centrifuged at 8000 rpm for 5 min. Once the phases were well-separated, the aqueous phase was separated and the hexane phase recovered and filtered with anhydrous sodium sulphate, which was rinsed two times with 2.5 mL of extraction buffer. The extraction procedure was repeated three times. The extract was further dried with a rotavapor at 30 °C and the obtained residue was dissolved in 5 mL of tetrahydrofuran (THF). The extracts were stored at −20 °C before analysis.

Spectrophotometric analysis was carried out to estimate lycopene content [43]. Measurements were performed with a spectrophotometer at 471 nm. Concentration (mg/kg fresh weight) was calculated by applying the molecular extinction coefficient of 0.365.

### 3.7. Phenolic Analysis

#### 3.7.1. Extraction for Assays

5 g of each of homogenised sample were extracted with 20 mL of methanol at ambient temperature for 30 min. The supernatant was collected, filtrated through a 0.45 μm membrane (Whatman International Ltd., Maidstone, UK) and diluted up to 25 mL in a volumetric flask with methanol.

#### 3.7.2. Total Phenolic (TP)

Total phenolic (TP) composition was determined with Folin Ciocalteau reagent from Sigma (St. Louis, MO, USA) using a Shimadzu UV 1601 spectrophotometer (Milan, Italy) [44].

Standard gallic acid solution (1000 ppm) was prepared dissolving 0.100 g of dry gallic acid in100 mL of a 80% methanol 20% water mixture using a 100 mL volumetric flask. 0, 50, 100, 200, 250 and 500 ppm calibration solutions were prepared by diluting the above solution with 80% methanol 20% water mixture.

From each calibration solution, methanol extracted sample, or blank, 100 μL were pipette into separate tubes and to each 6 mL of water, 1.5 mL of sodium carbonate solution (20% in water) and 500 μL of Folin-Ciocalteau reagent were added and well mixed.

The solutions were left at 20 °C for 2 h and the absorbance of each solution was determined at 725 nm against the blank (the 0 ppm calibration solution).

The total phenolic content was expressed as mg of gallic acid equivalents (GAE)/100 g of sample fresh weight.

#### 3.7.3. Individual Phenolic Compounds

Methanol extracted samples were filtrated through a 0.20 μm membrane and analyzed by HPLC in order to quantify each phenolic compound [45].

The analysis was performed with an Agilent 1100 Series HPLC system (Palo Alto, CA, USA) equipped with a quaternary pump (G13111A) and a diode array detector (G13114B) using a 20 μl sample injection loop. A reversed phase C18 column (150 × 4.6 mm i.d., particle size 5 μm; Agilent Eclipse XDB-C18) was employed. The eluents were 0.1% (*v*/*v*) formic acid in water (eluent A) methanol (eluent B). The gradient program was 0–50 % B in 50 min at a constant flow of 1 mL/min. The detection was at 325 nm for chlorogenic acid, 360 for rutin and quercitin. Identification was made by comparing retention times to those of commercially available standards purchased from Sigma (St. Louis, MO, USA).

Calibration curves were made for each standard using five different concentrations (1, 10, 20, 50 and 100 mg/L). A high linearity (R^2^ > 0.999) was obtained for each standard curve. All data are presented as mean ± standard deviation and expressed as mg/100 g of sample fresh weight.

### 3.8. Determination of the Organic Acids

The organic acid content was determined according to the method reported by Flores et al. [46] with slight modifications. Extracts were diluted to 30% *w*/*v* with deionised water, filtered through a 0.45 nm filter and submitted to high-performance liquid chromatography (HPLC) analysis. Citric, malic, glutamic, succinic, oxalic and ascorbic acids were identified by comparing their retention times to those of the standards (Sigma, St. Louis, MO, USA). The results were expressed as mg/100 g of fresh sample. The HPLC analysis was performed on a HPLC Agilent 1100 Series system equipped with a diode array detector using a 20 μl sample injection loop. UV detection was performed at 210 nm. A reversed-phase column, Spherisorb S5 ODS2 (5 μm, 250 × 4.6 mm; Waters Corporation, Milford, MA, USA), was used, and the elution was carried out under isocratic conditions using water acidified with orthophosphoric acid (pH 2.1) as the mobile phase at a flow rate of 0.6 mL/min for 45 min.

### 3.9. Volatile Organic Compounds by HS-SPME and GC/MS

SPME-GC/MS was used for sampling and analysis of the volatile organic compounds (VOC) in tomato samples [47]. A SPME holder containing a fused-silica fiber coated with a 50/30 μm layer of divinyl-benzene/carboxen/polydimethylsiloxane (DVB/CAR/PDMS) from Supelco (Bellafonte, PA, USA was used for the absorption of the volatile compounds.

Two grams of homogenized sample were weighed into a 20 mL headspace vial and added with 200 μL of 1-pentanol as internal standard (10 ppm).

The SPME fibre was exposed in the head space of the vial for 20 min at 45°C and then removed from the vial and introduced directly into the GC injector where the thermal desorption of the analytes was performed at 250 °C for 5 min.

A GC system 6890N equipped with a mass detector 5973 Agilent Technologies were used.

The analytes were separated on a 30 m × 0.250 mm capillary column coated with a 0.25 μm film of 5% diphenyl l95% dimethylpolysiloxane (HP5MS J&W Scientific, Folsom, CA, USA) and were inserted directly into the ion source of the mass detector. Splitless injection was used for the samples. The column oven temperature was programmed at 10 °C/min from an initial temperature of 50 (held for 2 min) to 150 °C, then at 15 °C/min to 300°C, which was held for 10 min. The injection and ion source temperatures were 250 and 230 °C, respectively. Helium (99.999%) was used as carrier gas at a flow rate of 1 mL/min. The ionizing electron energy was 70eV and the mass range scanned was 40–450 amu in full-scan acquisition mode [48].

The compounds were identified by comparing their retention indices and mass spectra with those found in the libraries NIST Atomic Spectra Database version 1.6. The VOC content were expressed as mg/kg of sample fresh weight.

### 3.10. Statistical Analysis

All determinations were performed in triplicate, and the reported results are the average values of the three repetitions. One-way analysis of variance (ANOVA) and Duncan’s multiple-range test (*p* ≤ 0.05) were conducted on the data using the software XLSTAT (Addinsoft, New York, NY, USA).

## 4. Conclusions

The chemical compositions of PdP and CIL cherry tomatoes were determined by means of fundamental chemical parameters, such as the main organic acids, carotenoids, flavonoids and organic volatile compounds. In this study, it has been demonstrated that the chemical profiles of antioxidants differ from one variety to the other and, hence, are variety-dependent, as is also the case for the majority of other parameters. Additionally, it has been shown that the concentration levels of the selected chemical parameters also varied as a function of the storage time. For the first time these two varieties have been compared taking into account several different physical and chemical markers and the influence of the storage on the quality of this product. The results presented and discussed herein can be useful for characterizing the health-promoting properties of these two tomato varieties and, consequently, improving the functional roles of these typical cultivars from the southern part of Italy (i.e., the Campania region) in the Mediterranean diet. These varieties have a great importance in the economy of the agricultural products of the Campania region, and this work could help the producers and also the retailers to have an orientation of some parameters that influence the quality of both products and also of how the storage conditions could be responsible of some important changes that are directly related with the sale of the final products.

## Figures and Tables

**Table 1:** pH, total soluble solids (TSS), dry matter (DM), reducing sugar, sodium chloride and titratable acidity (mean ± standard deviation) detected in tomato samples.

Sample	pH	TSS (°Brix)	DM (%)	Reducing Sugars (%)	Sodium Chloride (%)	Titratable Acidity (%Citric Acid)
**PdP 0**	4.36 ^a, b^ ± 0.04	6.9 ^c^ ± 0.1	7.20 ^c^ ± 0.19	2.89 ^c^ ± 0.07	0.18 ^b^ ± 0.01	0.53 ^a, b^ ± 0.01
**PdP 6**	4.45 ^a^ ± 0.01	7.9 ^b^ ± 0.1	8.40 ^b^ ± 0.34	2.00 ^d^ ± 0.11	0.20 ^b^ ± 0.02	0.50 ^a, b^ ± 0.04
**CIL 0**	4.29 ^b^ ± 0.01	6.3 ^d^ ± 0.1	7.71 ^b, c^ ± 0.38	3.77 ^b^ ± 0.10	0.16 ^b^ ± 0.04	0.43 ^b^ ± 0.05
**CIL 1**	4.13 ^c^ ± 0.06	8.5 ^a^ ± 0.1	9.55 ^a^ ± 0.12	4.12 ^a^ ± 0.05	0.41 ^a^ ± 0.04	0.57 ^a^ ± 0.03

a–c: different letters in the same column correspond to significant differences (*p* < 0.05).

**Table 2:** Colour readings and lycopene content (mean ± standard deviation) in tomato samples.

Sample	L	a	B	a/b	Hue	Lycopene (mg/kg Fresh Weight)
**PdP 0**	42.66 ^a^ ± 0.51	23.08 ^a^ ± 0.86	19.88 ^a^ ± 0.95	1.17 ^a^ ± 0.04	0.64 ^b^ ± 0.03	73.52 ^a^ ± 7.40
**PdP 6**	42.02 ^a^ ± 1.35	23.07 ^a^ ± 0.21	20.49 ^a^ ± 1.60	1.13 ^a^ ± 0.04	0.67 ^b^ ± 0.04	43.32 ^b^ ± 3.10
**CIL 0**	38.33 ^b^ ± 0.60	14.95 ^b^ ± 0.91	18.62 ^a^ ± 0.63	0.80 ^b^ ± 0.01	1.00 ^a^ ± 0.00	27.14 ^b^ ± 0.69
**CIL 1**	37.64 ^b^ ± 0.69	12.68 ^b^ ± 0.88	11.11 ^b^ ± 0,88	1.14 ^a^ ± 0.14	0.67 ^b^ ± 0.09	32.39 ^b^ ± 0.93

a–b: different letters in the same column correspond to significant differences (*p* < 0.05).

**Table 3:** Total phenolic (TP) compounds, rutin, quercitin and chlorogenic acid (mean ± standard deviation) in tomato samples.

Sample	TP (mg GAE/100 g Fresh Weight)	Rutin (mg/100 g Fresh Weight)	Quercitin (mg/100 g Fresh Weight)	Chlorogenic Acid (mg/100 g Fresh Weight)
**PdP 0**	38.544 ^a^ ± 0.07	7.37 ^b^ ± 0.01	0.95 ^b^ ± 0.01	10.39 ^a^ ± 0.66
**PdP 6**	34.27 ^b^ ± 0.11	8.98 ^a^ ± 0.15	0.34 ^b^ ± 0.01	7.34 ^b^ ± 0.83
**CIL 0**	29.631 ^c^ ± 0.97	4.41 ^d^ ± 0.01	2.18 ^a^ ± 0.03	7.94 ^b^ ± 1.66
**CIL 1**	37.483 ^a, b^ ± 0.19	5.63 ^c^ ± 0.01	1.87 ^a^ ± 0.03	7.16 ^b^ ± 0.10

a–c: different letters in the same column correspond to significant differences (*p* < 0.05).

**Table 4:** Mean and standard deviation of organic acids (mg/100 g fresh weight) content.

Sample	Malic Acid	Citric Acid	Succinic Acid	Oxalic Acid	Glutamic Acid	Ascorbic Acid
**PdP 0**	580.48 ^a^ ± 6.88	441.66 ^c^ ± 0.03	54.38 ^a^ ± 3.70	8.95 ^c^ ± 0.56	826.45 ^a^ ± 11.52	11.28 ^b^ ± 0.76
**PdP 6**	492.62 ^b^ ± 3.89	404.14 ^c^ ± 2.02	25.65 ^b^ ± 1.12	3.00 ^d^ ± 0.69	830.47 ^a^ ± 9.39	9.78 ^c^ ± 0.96
**CIL 0**	65.58 ^d^ ± 1.11	557.60 ^b^ ± 1.26	16.21 ^b^ ± 7.30	25.05 ^b^ ± 0.33	349.60 ^c^ ± 2.78	15.50 ^a^ ±0 .33
**CIL 1**	108.91 ^c^ ± 1.43	811.51 ^a^ ± 9.17	48.54 ^a^ ± 3.34	49.90 ^a^ ± 0.04	629.60 ^b^ ± 11.62	9.2 ^c^ ± 0.48

a–d: different letters in the same column correspond to significant differences (*p* < 0.05).

**Table 5:** VOC (mg/kg of fresh weight ± standard deviation) detected in samples in amount >1%.

Compounds	Samples			
	PdP 0	PdP 6	CIL 0	CIL 1
**Σ Aldheydes**	67.77 ^b^ ± 3.30	120.52 ^a^ ± 2.70	83.12 ^b^ ± 3.90	39.97 ^c^ ± 2.90
Pentanal	1.25 ^a^ ± 0.12	1.99 ^a^ ± 0.83	1.38 ^a^ ± 0.11	1.46 ^a^ ± 0.02
Hexanal	55.79 ^b^ ± 2.13	100.22 ^a^ ± 2.70	57.22 ^b^ ± 1.60	25.90 ^c^ ± 0.70
Heptanal	4.27 ^a^ ± 0.11	0.49 ^c^ ± 0.06	3.19 ^a, b^ ± 0.50	2.35 ^b^ ± 0.55
Octanal	0.74 ^c^ ± 0.15	0.66 ^c^ ± 0.70	3.56 ^a^ ± 0.70	1.74 ^b^ ± 0.50
Decanal	0.25 ^a^ ± 0.11	0.32 ^a^ ± 0.09	1.19 ^a^ ± 0.56	0.47 ^a^ ± 0.04
2-Hexenal (*E*)	4.83 ^b^ ± 0.03	14.64 ^a^ ± 0.03	13.94 ^a^ ± 3.70	5.39 ^b^ ± 0.70
2-Heptenal (*Z*)	0.50 ^a^ ± 0.26	0.94 ^a^ ± 0.05	1.31 ^a^ ± 0.55	1.18 ^a^ ± 0.67
**Σ Alcohols**	59.32 ^a^ ± 0.90	17.48 ^b^ ± 1.90	23.22 ^b^ ± 2.70	25.39 ^b^ ± 2.70
2,4-Hexandien-1-ol	0.01 ^c^ ± 0.09	0.64 ^b^ ± 0.07	1.11 ^b^ ± 0.07	4.02 ^a^ ± 0.03
3-Hexen-1 ol (*Z*)	13.52 ^a^ ± 0.70	7.72 ^b^ ± 0.66	14.30 ^a^ ± 0.13	13.78 ^a^ ± 0.16
1-Hexanol	44.80 ^a^ ± 0.19	9.13 ^b^ ± 0.02	7.27 ^b^ ± 0.08	6.43 ^b^ ± 0.18
**Σ Ketones**	1.25 ^b^ ± 0.14	1.40 ^b^ ± 0.70	2.75 ^b^ ± 0.22	3.65 ^a^ ± 0.11
6-Methyl-5-hepten-2-one	1.25 ^b^ ± 0.22	1.40 ^b^ ± 0.24	2.75 ^a, b^ ± 0.44	3.38 ^a^ ± 0.02
**Σ Acids**	0.58 ^b^ ± 0.27	3.77 ^a^ ± 0.04	1.88 ^b^ ± 0.09	0.35 ^b^ ± 0.23
Acetic acid	0.15 ^b^ ± 0.09	2.79 ^a^ ± 0.27	0.01 ^b^ ± 0.08	0.02 ^b^ ± 0.02
Butanoic acid	0.13 ^a^ ± 0.01	0.58 ^a^ ± 0.01	1.29 ^a^ ± 0.01	0.02 ^a^ ± 0.01
**Σ Others**	34.66 ^a^ ± 0.10	7.86 ^c^ ± 0.02	1.76 ^c^ ± 0.01	15.49 ^b^ ± 0.08
Propane	4.70 ^b^ ± 0.04	2.08 ^b, c^ ± 0.04	0.02 ^c^ ± 0.04	13.19 ^a^ ± 0.01
3-Methyl-2,4-hexadiene	0.02 ^b^ ± 0.01	1.02 ^a^ ± 0.01	0.02 ^b^ ± 0.01	0.02 ^b^ ± 0.01
2-Isobutylthiazole	29.44 ^a^ ± 0.01	3.34 ^b^ ± 0.01	0.02 ^b^ ± 0.01	0.02 ^b^ ± 0.01

a–c: different letters in the same row correspond to significant differences (*p* < 0.05).
